# Imaging Characteristics of Hemangiopericytoma: A Case Report

**DOI:** 10.7759/cureus.92912

**Published:** 2025-09-22

**Authors:** Neelam Katre, Harini Bopaiah, Anil K Sakalecha, Anees Dudekula

**Affiliations:** 1 Radiodiagnosis, Sri Devaraj Urs Academy of Higher Education and Research (SDUAHER), Kolar, IND

**Keywords:** dural-based lesion, hemangiopericytoma (hpc), intracranial tumor, nab2–stat6 gene fusion, solitary fibrous tumor (sft)

## Abstract

Hemangiopericytoma (HPC) is an uncommon, dural-based, highly vascular intracranial tumor that may mimic meningioma on imaging. These lesions often present with mass effect symptoms and can demonstrate aggressive behavior, including local invasion and recurrence. In this report, we present the case of a 62-year-old female who experienced occipital headaches and was found to have a dural-based lesion in the posterior fossa on MRI. Imaging revealed features including isointensity to hypointensity on T1-weighted images, heterogeneous signal on T2/fluid-attenuated inversion recovery (FLAIR), absence of diffusion restriction, blooming on susceptibility-weighted imaging (SWI), and heterogeneous contrast enhancement with necrotic areas. Unlike meningioma, the lesion showed more aggressive imaging characteristics, including necrosis and prominent vascularity, features more suggestive of HPC. Neurosurgical evaluation and histopathology were essential for diagnosis and further management. The patient underwent left posterior fossa craniotomy with gross total resection of a well-circumscribed, highly vascular dural-based mass. Histopathology confirmed WHO Grade II HPC. Due to the tumor's aggressive nature, adjuvant fractionated radiotherapy was initiated within six weeks. The patient remained neurologically stable on follow-up and was counseled on the need for long-term surveillance.

## Introduction

Hemangiopericytomas (HPCs) are rare, aggressive mesenchymal tumors of pericytic origin, now classified under solitary fibrous tumors (SFTs) in the 2021 WHO Classification of Tumors of the Central Nervous System due to their shared histological and genetic features. Clinically, patients may present with symptoms related to the mass effect, such as headaches, focal neurological deficits, or seizures, depending on the tumor’s location [1]. Notably, the NAB2-STAT6 gene fusion is a key diagnostic marker for HPC [2].

They account for less than 1% of all intracranial tumors and primarily affect middle-aged adults, with a slight male predominance [3].

While HPCs often mimic meningiomas on imaging, magnetic resonance imaging (MRI) can reveal distinguishing features such as heterogeneous enhancement, necrosis, and flow voids on susceptibility-weighted imaging (SWI), which assist in preoperative differentiation [4,5]. Histologically, they exhibit a staghorn vascular pattern and express markers including CD34 and STAT6, helping to distinguish them from other spindle cell tumors [6].

Due to their high recurrence and metastatic potential, gross total resection followed by long-term surveillance is critical. Postoperative radiotherapy may be considered in select cases to reduce recurrence risk [7,8].

## Case presentation

A 62-year-old female patient presented with complaints of persistent headache localized to the occipital region, which had gradually worsened over the preceding 10 days. The headache was described as dull and non-pulsatile, with no associated nausea, vomiting, photophobia, or neurological deficits. The patient denied any history of recent trauma, seizures, loss of consciousness, or visual disturbances. There was no significant past medical or surgical history, and she was not on any long-term medications. General and neurological examinations were unremarkable, and her vital signs were stable.

Table 1 describes the difference between HPC and meningioma.

**Table 1:** Difference between hemangiopericytoma and meningioma

Feature	Meningioma	Hemangiopericytoma (HPC)
Location	Dural-based, extra-axial	Dural-based, extra-axial
Patient demographics	More common in middle-aged and elderly adults	More common in middle-aged and older adults
Shape and margins	Well-circumscribed, smoothly marginated	Irregular or lobulated margins, less defined
Enhancement pattern	Homogeneous enhancement	Heterogeneous enhancement with necrotic/cystic areas
Dural tail sign	Frequently present	Rare or absent
Bone involvement	Often causes hyperostosis	May cause bone erosion or destruction
Edema	Minimal or mild peritumoral edema	Often extensive surrounding edema
Calcifications	Common	Rare
Flow voids/vascularity	Typically less vascular	Highly vascular with prominent flow voids
Growth pattern	Slow-growing, benign	Aggressive, invasive, higher recurrence
Metastasis	Rare	Possible, including distant spread
MRI utility	Supports diagnosis but not definitive	Suggestive but requires histopathology for confirmation
Histopathological requirement	Usually supportive, but not always necessary	Always required for definitive diagnosis

Imaging features of our case

**Figure 1:**
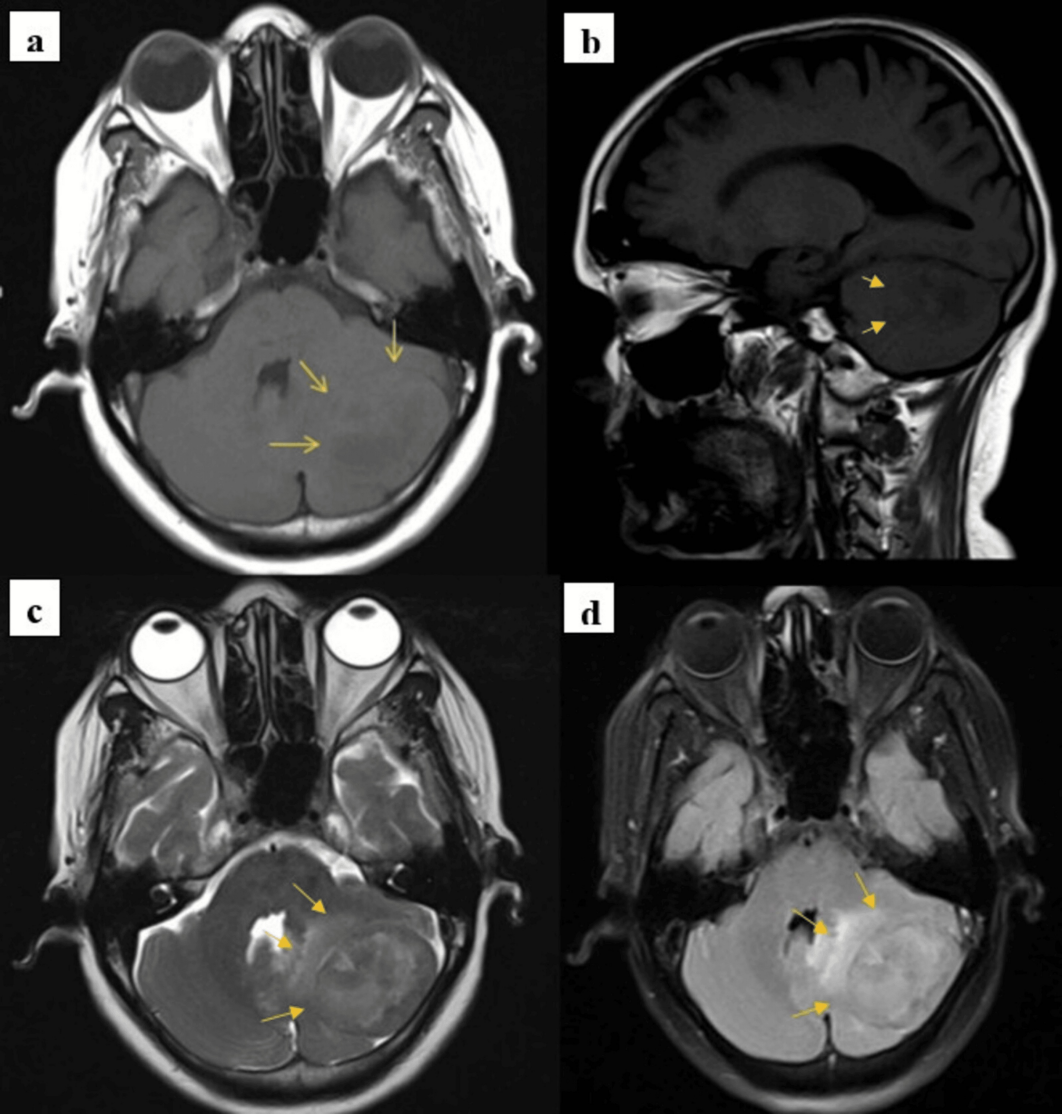
(a) MRI T1 axial section showing a large, well-defined, ovoid, extra-axial isointense to hypointense lesion in the left posterior fossa (yellow arrow). (b) MRI T1 sagittal section showing a large, well-defined, ovoid, extra-axial isointense to hypointense lesion in the left posterior fossa (yellow arrow). (c,d) T2/fluid-attenuated inversion recovery (FLAIR) images showing a hypointense-hyperintense lesion in the left posterior fossa with a broad base toward the dura (tentorium). The lesion is causing mass effect with compression of the adjacent cerebellar hemisphere, brainstem, and fourth ventricle.

Axial, sagittal T1-weighted MRI sequences revealed a large, well-defined, ovoid extra-axial lesion situated in the left posterior cranial fossa. The lesion appeared isointense to hypointense on T1 images and was noted to have a broad base of attachment to the dura along the tentorium cerebelli. On T2-weighted and fluid-attenuated inversion recovery (FLAIR) sequences, the lesion demonstrated hypointense to hyperintense signal characteristics. There was marked mass effect, evidenced by compression of the adjacent cerebellar hemisphere, indentation of the brainstem, and partial effacement of the fourth ventricle, as shown in Figure 1.

**Figure 2:**
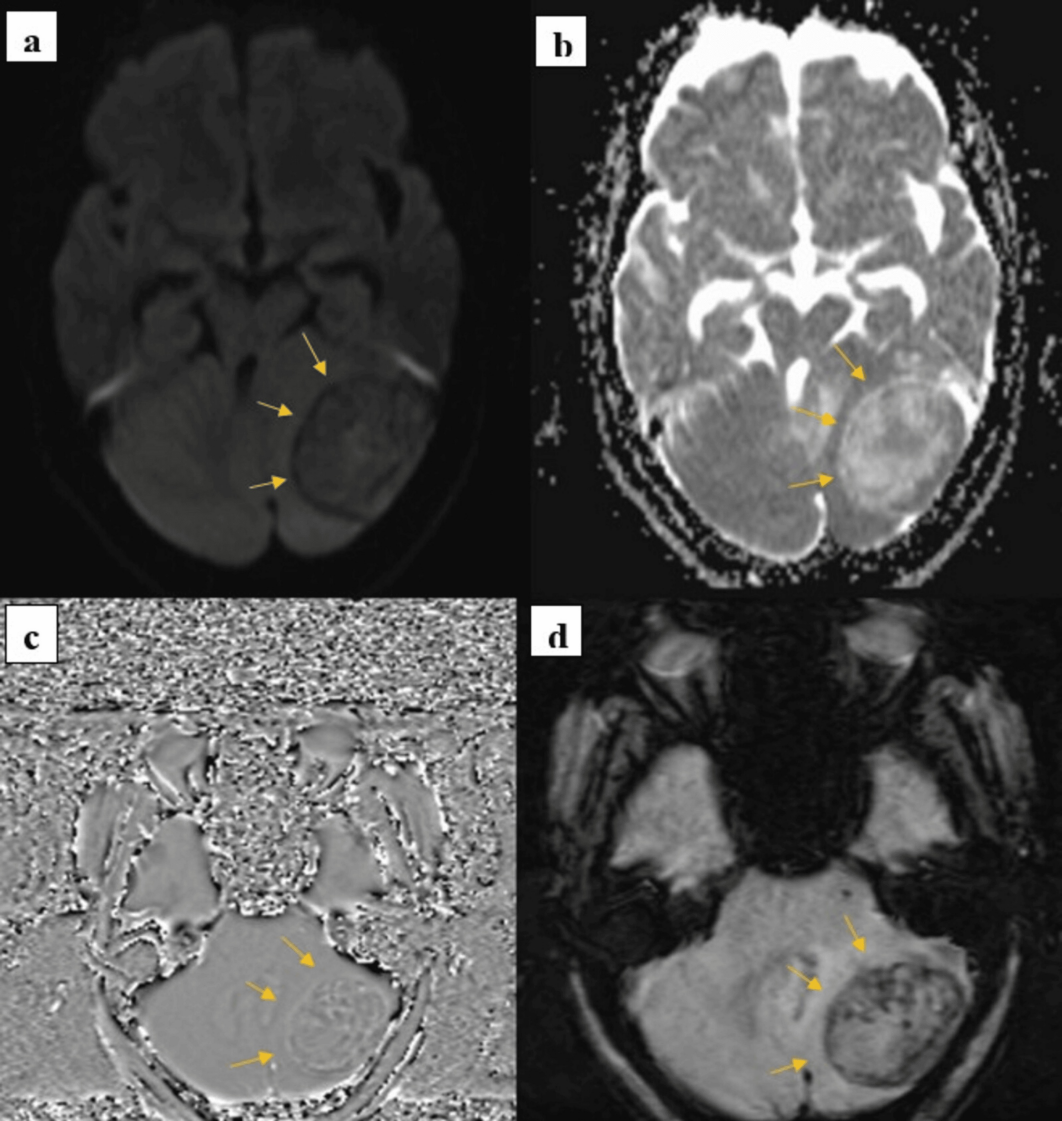
(a) Diffusion-weighted imaging (DWI) and (b) apparent diffusion coefficient (ADC): The lesion shows no evidence of restricted diffusion on DWI (yellow arrows). (c,d) A few blooming foci are noted on susceptibility-weighted imaging (SWI) within the lesion (yellow arrow), suggestive of calcifications.

Diffusion-weighted imaging (DWI) showed no evidence of restricted diffusion within the lesion, reducing the likelihood of an acute infarct or high-grade cytotoxic lesion. SWI revealed a few blooming foci within the lesion, suggestive of calcification components, as shown in Figure 2.

**Figure 3:**
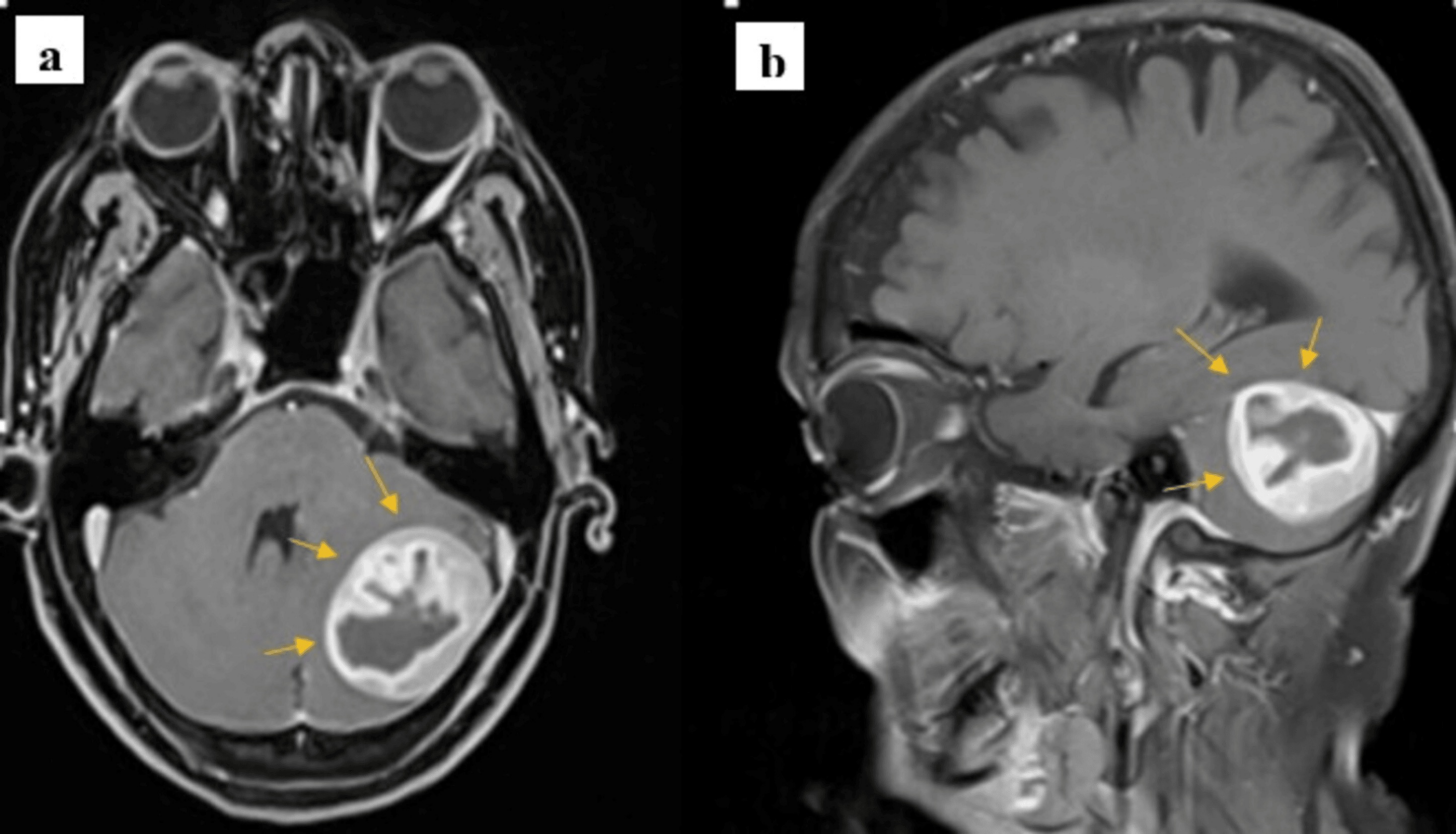
(a,b) Axial and sagittal post-contrast MRI demonstrates avid heterogeneous peripheral enhancement (yellow arrow) with central non-enhancing areas, likely necrotic. Adjacent dural enhancement (dural tail) is noted. Mild adjacent peripheral edema is also present.

Post-contrast T1-weighted images demonstrated avid, heterogeneous peripheral enhancement with central non-enhancing areas suggestive of necrosis. There was also mild adjacent dural enhancement (dural tail sign) and surrounding cerebellar edema, which further supported the suspicion of a dural-based neoplasm, as shown in Figure 3.

Based on the clinical presentation and characteristic imaging features, a preliminary radiological diagnosis of HPC was made. The patient was advised to undergo neurosurgical evaluation and histopathological correlation to confirm the diagnosis and plan further management.

Following, the patient was referred for neurosurgical evaluation and recommended surgical excision, and a left posterior fossa craniotomy was performed, achieving gross total resection of a well-circumscribed, highly vascular dural-based mass. Histopathology confirmed WHO Grade II HPC. Given the tumor’s aggressive nature, adjuvant fractionated radiotherapy was initiated within six weeks postoperatively. The patient was placed on a structured follow-up plan. Patient remained neurologically stable, and counseling was provided regarding the need for long-term surveillance.

## Discussion

HPCs, now reclassified under SFTs according to the 2021 World Health Organization classification of central nervous system tumors, are rare mesenchymal neoplasms arising from Zimmermann’s pericytes - specialized contractile cells surrounding capillaries and venules [1,2].

These tumors constitute less than 1% of all intracranial neoplasms and typically affect adults between the third and sixth decades of life, as reported by Ashizawa et al. [3]. Our case aligns with this demographic pattern, presenting in a middle-aged patient with non-specific neurological symptoms.

Radiologically, HPCs often mimic meningiomas due to their dural-based, extra-axial location. However, subtle imaging differences are crucial for accurate diagnosis. MRI typically reveals an extra-axial mass that appears isointense to gray matter on T1-weighted images, isointense to hyperintense on T2-weighted images, and demonstrates intense, heterogeneous post-contrast enhancement owing to its rich vascularity. In our patient, these features were clearly evident and consistent with findings described by Xiong et al. [4].

Ma et al. [5] emphasized the diagnostic significance of identifying features such as flow voids, irregular tumor margins, and prominent vascular channels - hallmarks that assist in differentiating HPCs from more benign-appearing meningiomas.

Histopathological examination remains essential, typically revealing tightly packed spindle cells with a characteristic “staghorn” vascular pattern. Immunohistochemistry shows positivity for CD34, STAT6, and vimentin - features that support a diagnosis of HPC/SFT and help differentiate it from other spindle cell tumors, as described by Yoshida et al. [6]. In our case, these histological and immunophenotypic findings were confirmed.

Surgical resection is the cornerstone of treatment. However, HPCs are associated with high rates of recurrence and extracranial metastasis, up to 25% and 68% respectively - necessitating long-term imaging surveillance [7]. In our case, gross total resection was achieved without immediate complications. Adjuvant radiotherapy remains controversial but is often recommended for subtotal resections or higher-grade tumors, as it may improve local control [8].

## Conclusions

Intracranial HPC, although rare, should be considered in the differential diagnosis of dural-based, extra-axial brain lesions, especially in middle-aged and older adults. While MRI is invaluable for early detection and surgical planning, demonstrating features such as heterogeneous enhancement, necrotic components, and a broad dural attachment, it cannot definitively diagnose HPC. Histopathological confirmation remains essential for accurate diagnosis and guiding treatment decisions. Given the tumor’s aggressive nature, high recurrence rates, and potential for distant metastasis, radiologists and clinicians should maintain a high index of suspicion and implement rigorous long-term surveillance protocols to optimize patient outcomes.
